# Differentiation of Taxonomically Closely Related Species of the Genus *Acinetobacter* Using Raman Spectroscopy and Chemometrics

**DOI:** 10.3390/molecules24010168

**Published:** 2019-01-04

**Authors:** A. Margarida Teixeira, Alexandr Nemec, Clara Sousa

**Affiliations:** 1 LAQV/REQUIMTE, Departamento de Ciências Químicas, Faculdade de Farmácia, Universidade do Porto, Rua Jorge Viterbo Ferreira, 228, 4050-313 Porto, Portugal; up201404495@ff.up.pt; 2 Laboratory of Bacterial Genetics, Centre for Epidemiology and Microbiology, National Institute of Public Health, Šrobárova 48, 100 42 Prague 10, Czech Republic; anemec@szu.cz; 3 Department of Laboratory Medicine, Third Faculty of Medicine, Charles University, Šrobárova 50, 100 34 Prague 10, Czech Republic

**Keywords:** vibrational spectroscopy, bacteria, typing, species, haemolytic clade

## Abstract

In recent years, several efforts have been made to develop quick and low cost bacterial identification methods. Genotypic methods, despite their accuracy, are laborious and time consuming, leaving spectroscopic methods as a potential alternative. Mass and infrared spectroscopy are among the most reconnoitered techniques for this purpose, with Raman having been practically unexplored. Some species of the bacterial genus *Acinetobacter* are recognized as etiological agents of nosocomial infections associated with high rates of mortality and morbidity, which makes their accurate identification important. The goal of this study was to assess the ability of Raman spectroscopy to discriminate between 16 *Acinetobacter* species belonging to two phylogroups containing taxonomically closely related species, that is, the *Acinetobacter baumannii*-*Acinetobacter calcoaceticus* complex (six species) and haemolytic clade (10 species). Bacterial spectra were acquired without the need for any sample pre-treatment and were further analyzed with multivariate data analysis, namely partial least squares discriminant analysis (PLSDA). Species discrimination was achieved through a series of sequential PLSDA models, with the percentage of correct species assignments ranging from 72.1% to 98.7%. The obtained results suggest that Raman spectroscopy is a promising alternative for identification of *Acinetobacter* species.

## 1. Introduction

Vibrational spectroscopy encompasses a group of spectroscopic techniques which are based on the molecular vibrations of a certain sample. These techniques (mostly infrared and Raman) have been explored in the context of bacterial typing [1,2], with several works published reporting different degrees of success depending on the technique, the bacteria themselves, and/or the taxonomic levels [3,4,5,6]. Some studies also suggest that vibrational spectroscopy could have similar or even greater potential to be used routinely for bacterial typing than already recognized mass spectrometry-based techniques such as matrix assisted laser desorption ionization time-of-flight mass spectrometry (MALDI-TOF MS) [7,8]. Among vibrational spectroscopic techniques, Raman spectroscopy has gathered less attention for typing proposes and most published studies are deficient in the number of bacterial isolates and/or genus, species, or strains included [4,6,9]. Also, despite some effort made towards standardization, little is known about how culture media, time of growth, cell handling prior to spectra acquisition, or equipment settings (such as laser power, time of exposure, and spectra accumulations) influence the final result. With this in mind, the aim of the present study was to assess the ability of Raman spectroscopy to discriminate between bacterial species which are phylogenetically so closely related that their differentiation using conventional methods can be problematic. To achieve this goal, 16 species belonging to two well-defined phylogroups of the *Acinetobacter* genus were selected. This genus accommodates Gram-stain-negative and strictly aerobic organisms, which live in diverse ecosystems, with some of them being important opportunistic pathogens in humans [10]. The first phylogroup is the *Acinetobacter calcoaceticus*-*Acinetobacter baumannii* (ACB) complex, which currently contains six species with validly published names [11,12,13]. The ACB complex includes *A. baumannii*, medically the most important *Acinetobacter* species, and *Acinetobacter pittii*, which is the most prevalent species of the genus in non-epidemic situations. The second phylogroup is the so-called haemolytic clade which typically, although not exclusively, encompasses species displaying strong haemolytic activity on agar media such as *Acinetobacter beijerinckii*, *Acinetobacter colistiniresistens*, *Acinetobacter haemolyticus*, and *Acinetobacter proteolyticus* [14,15]. Members of each of these phylogroups have been variously associated with several identification issues at the species level, even when using molecular techniques such as whole cell profiling based on MALDI-TOF MS [16,17] which over-justify additional efforts aimed at accurate and rapid identification. In this work, a series of partial least squares discriminant analysis (PLSDA) models were developed, subsequently leading to a flowchart enabling *Acinetobacter* species discrimination. The corresponding confusion matrices lead to 72.1–98.7% accuracy in species prediction, which is quite comparable with results obtained by mass or infrared spectroscopic techniques. This work could have benefitted from introducing a higher number of isolates of each species and/or additional species to strengthen the results. However, the results herein obtained proved the potential of Raman spectroscopy in the context of bacterial species identification. Also, to the best of our knowledge, *Acinetobacter* species discrimination through Raman spectroscopy has never been attempted with most of the species included in our work, or with such a high number of species or strains.

## 2. Results

### 2.1. Spectral Analysis

**Figure 1:**
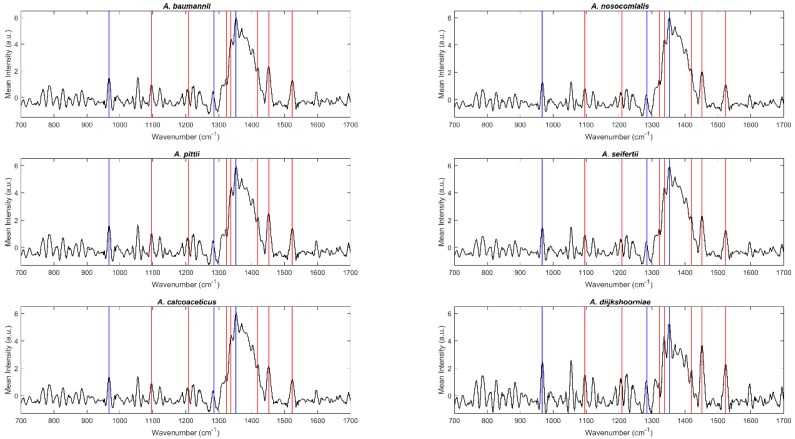
Mean Raman spectra of *Acinetobacter spp.* belonging to the *Acinetobacter baumannii*-*Acinetobacter calcoaceticus* complex. Blue and red vertical lines correspond to peaks attributed to agar and bacterial component vibrations, respectively.

**Figure 2:**
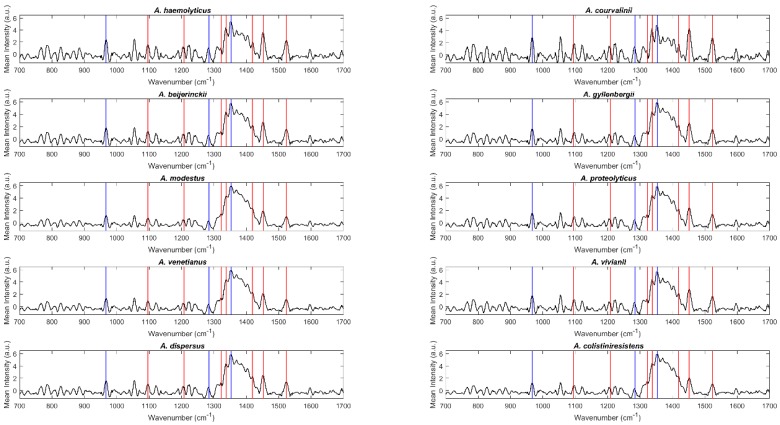
Mean Raman spectra of *Acinetobacter spp.* belonging to the *Acinetobacter* haemolytic clade. Blue and red vertical lines correspond to peaks attributed to agar and bacterial component vibrations, respectively.

Raman spectra were acquired between 100 cm^−1^ and 2300 cm^−1^ for all bacterial isolates included in this work (details about replicates are included in the Section 4.2 Raman spectroscopy). Figure 1 and Figure 2 include the mean spectra (700 cm^−1^ and 1700 cm^−1^) obtained for each species, namely, species of the ACB complex and the haemolytic clade, respectively. Overall, the spectra present the typical patterns of a bacterial Raman spectrum and are quite similar among the analyzed species, demanding a chemometric analysis for species discrimination. Blue vertical lines in Figure 1 and Figure 2 correspond to peaks usually attributed to agar vibrations, with the red ones due to the bacterial components. Peaks at 966 cm^−1^, 1285 cm^−1^ and 1350 cm^−1^ (blue lines) are commonly attributed to the deformation, twisting, and wagging of the agar molecules [18]. The region between 1030 and 1130 cm^−1^ of a Raman bacterial spectrum is recognized as reflecting carbohydrate vibrations (mostly C–C, C–O, and C–O–H). Regarding the peaks marked as belonging to bacteria (red lines), peaks at 1096 cm^−1^ were assigned to nucleic acids and glycosidic linkages of saccharides vibrations, at 1209 and 1323 cm^−1^ to amide III (C–C stretching and C–N + N–H stretching, respectively), at 1336 cm^−1^ and 1452 cm^−1^ to amide III N–H stretching plus CH_2_ and CH_3_ fatty acid deformation, at 1421 cm^−1^ to −C–O vibrations of peptidoglycan, and at 1523 cm^−1^ to −C–C conjugated stretching [18].

### 2.2. Discrimination of Acinetobacter Species

**Figure 3:**
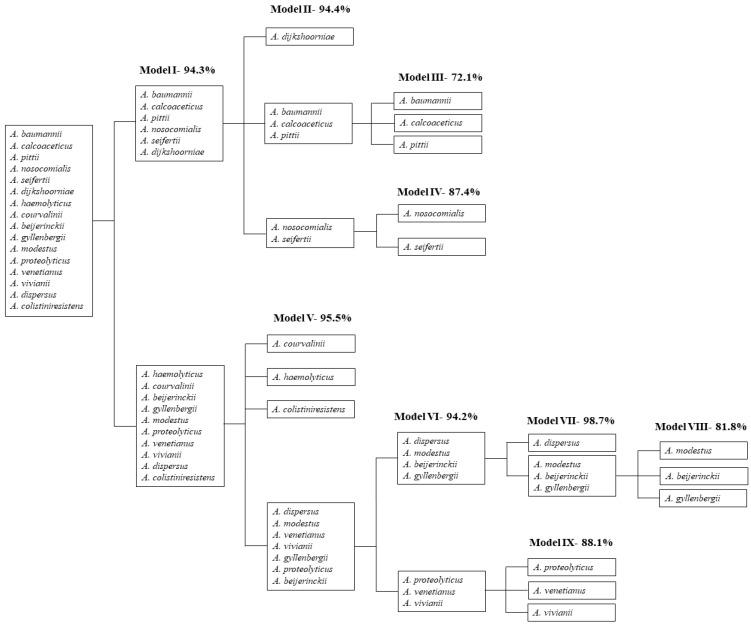
Flowchart developed for the identification of *Acinetobacter* species, including the number of developed partial least squares discriminant analysis (PLSDA) models and percentage of correct species assignments of each model.

Due to the large number of high similarity *Acinetobacter* species included in this study (Table S1), it was unfeasible to develop a single chemometric model able to discriminate between all species simultaneously. Species discrimination was undertaken through a sequence of chemometric models (PLSDA) according to the flowchart shown in Figure 3. The percentage of correct species prediction (Table 1, Table 2 and Table 3) from each model was obtained from the corresponding confusion matrix. It should be noted that this percentage corresponds solely to the validation set isolates, which strengthens the obtained results (for details, see Section 4.3. Data analysis). The species included in each discrimination level were selected through the development of unsupervised principal component analysis (PCA) models prior to all PLSDA models (data not shown). The PCA models, which were totally independent of the sample classes (in this case, the *Acinetobacter* species) revealed natural classes within the samples, indicating their relative similarity and dissimilarity. The first PCA model to be developed, which encompassed all the *Acinetobacter* species of this study, revealed two clusters, one containing the ACB complex species and the other containing the haemolytic clade species. Based on this finding, the first PLSDA model was developed, which aimed to discriminate between these two clusters. Following this, the same procedure was adopted repeatedly, allowing for a selection of species to be identified by each model (the flow of discrimination).

The first PLSDA model developed, Model I (Table 1), allowed for the discrimination of species of the ACB complex from those remaining (with a correct prediction rate of 94.3%). Discrimination among the ACB complex species was achieved through the development of three additional models (Table 2): Model II (*Acinetobacter dijkshoorniae*/*Acinetobacter nosocomialis* + *Acinetobacter seifertii*/*A. baumannii* + *A. calcoaceticus* + *A. pittii* (94.4%)), Model III (*A. baumannii*/*A. calcoaceticus*/*A. pittii* (72.1%)) and Model IV (*A. nosocomialis*/*A. seifertii* (87.4%)). Model V endeavored to identify the haemolytic clade species (Table 3), and four well defined clusters were observed in the PLSDA scores map. Three of them comprised isolates from a single species which enabled their discrimination, namely *Acinetobacter courvalinii*, *A. haemolyticus*, and *A. colistiniresistens*. The fourth cluster (Cluster BI, Table 3) encompassed the remaining seven species that were further modelled. The percentage of correct cluster/species assignments was 95.5%. The subsequent PLSDA model (Model VI) revealed two clusters, BI-A and BI-B (Table 3), which contained four and three *Acinetobacter* species respectively, with a percentage of correct assignments of 94.2%. This model allowed for the splitting of species into clusters which were further modelled (Model VII and Model IX). Regarding Cluster BI-A, *Acinetobacter dispersus* was discriminated from *Acinetobacter modestus*, *A. beijerinckii*, and *Acinetobacter gyllenbergii* (Model VII), with 98.7% of assignments correct. These three species were then distinguished (Model VIII), with 81.1% of species predictions correct. Cluster BI-B isolates (*A. proteolyticus*, *Acinetobacter venetianus*, and *Acinetobacter vivianii*) were then identified (Model IX), with a percentage of correct species predictions of 88.1%.

## 3. Discussion

In this work, 16 *Acinetobacter* species were distinguished though a combined Raman spectroscopy and chemometrics approach. Nine consecutive PLSDA models were undertaken with percentages of correct species discrimination ranging from 72.1% to 98.7% (results solely from the validation set isolates). To the best of our knowledge, no previous attempts to discriminate *Acinetobacter* isolates at the species level have been performed with Raman spectroscopy using such a comprehensive bacterial collection. Even considering other bacterial species and/or taxonomic levels, this technique has been barely explored, and most published studies have encompassed few isolates and/or species or isolates from different genera. In 2010, Paret and colleagues [19] proved the suitability of micro-Raman in discriminating Gram-stain-positive from Gram-stain-negative bacteria. Berger and Zhu [20] distinguished between four *Streptococcus* species (*Streptococcus mutans*, *Streptococcus sanguis*, *Streptococcus intermedius*, and *Streptococcus oralis*) using Raman microspectroscopy, with 93.8% of species assignments correct. It should be noted that these results were from the calibration set samples and no external model validations were undertaken. In 2013, van de Vossenberg and colleagues [4] successfully discriminated between *Escherichia coli*, *Legionella*, and several coliform isolates using Raman spectroscopy. However, their species discrimination results arose from isolates of distinct genera, which could have positively skewed the results. Other studies have been published aiming at species discrimination using Raman spectroscopy with the aid of more complex approaches, namely with nanocolloidal particles [21] and/or with surface enhanced Raman spectroscopy (SERS) [21,22]. Regarding *Acinetobacter* species, published works have included few *Acinetobacter* isolates, and none have directly explored species discrimination within the genus. Maquelin et al. [9] used Raman spectroscopy to discriminate between *A. baumannii*, *A. nosocomialis* (in that study termed *Acinetobacter* gen. sp. 13TU), and *A. pittii* (termed *Acinetobacter* gen. sp. 3) from different hospital outbreaks, without exploring species discrimination. Indeed, in that study, hierarchical cluster analysis of Raman spectra clustered isolates according to the outbreak but not with their species. Concerning competitive spectroscopic techniques, ACB complex species have been the target of most previous studies due to their clinical relevance, whereas the remaining species included in this work have been neglected. Sousa and colleagues [5] discriminated between ACB complex species by Fourier transform infrared spectroscopy (FTIR) and a series of PLSDA models, with 100% of species assignments correct for the test isolates. Most of the same isolates were discriminated by MALDI-TOF MS [23], also with 100% of species predictions correct, and with a consideration of intact cells spectra. It should be noted that the results obtained with MALDI-TOF MS were not from an independent test set of isolates but from a cross-validation set. Despite being quite poor, the results obtained herein regarding the ACB complex species (with 72.1–94.4% of species assignments correct) using Raman spectroscopy are promising. Further works that could contribute to the standardization of the technique (through a consideration of culture media, time of growth and/or equipment setting related parameters) will certainly lead to better results and accuracy in species discrimination. Nine PLSDA models were needed to discriminate the 16 *Acinetobacter* species included in this study. Such models, besides allowing for species discrimination, also revealed some insights into species similarity and dissimilarity within the genus. Of note, the first PLSDA model allowed for discrimination between the ABC complex species and those belonging to the haemolytic clade, which is in agreement with the results obtained from the core genome-based tree of the *Acinetobacter* genus presented by Nemec et al. [24]. Within the ABC complex, *A. nosocomialis* was grouped with *A. seifertii*, also in accordance with the results obtained by Nemec et al. (2018). However, *A. pittii* isolates were grouped with *A. baumannii* and *A. calcoaceticus* instead of being grouped with *A. dijkshoorniae*. The same was verified for the haemolytic clade isolates; some likely clustered in the Nemec et al. [24] study but most of them clustered randomly. It should be noted that species similarity and dissimilarity presented in the Nemec work was based on the amino acid sequence evolution of the species and the results herein obtained were based on their vibrational spectra, which could justify the differences encountered among clusters. Regarding the percentages of correct species assignments (72.1–98.7%), they are satisfactory overall, with the great majority near or above 90%. The exceptions were associated with models that aimed to discriminate between *A. baumannii*, *A. calcoaceticus*, and *A. pittii* (Model III), and between *A. beijerinckii*, *A. gyllenbergii*, and *A. modestus* (Model VIII). These two models presented lower percentages of correct species assignments (72.1% and 81.8%, respectively) which could be related to a higher species similarity and also to the fact that both models aimed to discriminate between three species at a time. It should be noted that the species included in each model were selected based on natural clusters encountered by the PCA models, and to reduce and/or change them certainly would have biased the results. These results seem to indicate that Raman spectroscopy could be a reliable technique for species discrimination, rather than it being useful to stablish phylogenetic relations among the *Acinetobacter* genus.

## 4. Material and Methods

### 4.1. Bacterial Collection

The study collection included 106 strains belonging to 16 *Acinetobacter* species (Table S1). Forty-three strains fell into six species of the ACB complex (*A. baumannii* (n = 7), *A. calcoaceticus* (n = 8), *A. dijkshoorniae* (n = 5), *A. nosocomialis* (n = 7), *A. pittii* (n = 8), and *A. seifertii* (n = 8)), whereas 63 strains were members of the haemolytic clade (*A. beijerinckii* (n = 6), *A. colistiniresistens* (n = 8), *A. courvalinii* (n = 7), *A. dispersus* (n = 6), *A. gyllenbergii* (n = 7), *A. haemolyticus* (n = 8), *A. modestus* (n = 6), *A. proteolyticus* (n = 6), *A. venetianus* (n = 5), and *A. vivianii* (n = 4)). The strains were selected either from previous taxonomic studies conducted by the Laboratory of Bacterial Genetics [12,13,14,15] or the study of Cosgaya and colleagues [11]. Details about the collection are in Table S1.

### 4.2. Raman Spectroscopy

Bacterial isolates were grown on two distinct days on TSA for 18 h at 37 °C (biological replicates). The generated biomass of each isolate was directly transferred from the agar plate to a regular microscope slide, making a ~5 mm diameter spot. Each spot was measured in three distinct locations (instrumental replicates), producing a total of six spectra per isolate. Raman spectra were acquired using a Raman CORA5700 (Cora 5X000 Raman spectrometers, Anton Paar, Ashland, VA, USA) equipped with a two excitation wavelengths laser (785 and 1064 nm). Spectra were collected with an excitation wavelength of 785 nm with a laser power of 140 mW over 1s and with a background signal of between 100–2300 cm^−1^.

### 4.3. Data Analysis

Due to the large number of species included in this work, it was not possible to distinguish them successfully using a single chemometric model. Instead, several models were developed to achieve species discrimination, namely partial least squares discriminant analysis models. The PLSDA model, which is based on PLS regression, requires previous knowledge of assigned classes (in this work, the *Acinetobacter* species or groups of species) for all tested isolates [25,26]. The classes of each PLSDA model were defined through exploratory principal component analysis models. Prior to each PLSDA model, one PCA model was developed in order to search for possible clusterization. It should be noted that the clusterization encountered in the PCA models did not depend on the sample classes and only reflected spectra similarity/dissimilarity. The number of clusters found in each PCA model was used to define the classes (species) to be modelled by PLSDA. More information regarding PLSDA model optimization and its application in a similar context has been described elsewhere [27]. Prior to modelling, spectra were divided into two data sets (70% for calibration and 30% for validation) and pre-processed with standard normal variate (SNV) followed by the application of a Savitzky-Golay (SavGol) filter (where x is filter width, y is polynomial order, and z is derivative order) and mean-centered. The pre-processing technique and the number of latent variables (LVs) were selected for each developed model based on the higher percentage of correct species classifications obtained through the confusion matrix of the corresponding PLSDA model. It should be noted that the PLSDA confusion matrix reflects solely the percentage of correct species predictions of the validation set. All calculations were carried out using Matlab version 8.6 (R2015b) (Mathworks, Natick, MA, USA) and the PLS Toolbox version 8.1 for Matlab (Eigenvector Research, Manson, WA, USA).

## 5. Conclusions

In this work, 16 phylogenetically close *Acinetobacter* species were distinguished from one another by Raman spectroscopy, with percentages of correct assignments ranging from 72.1–98.7%. The results herein obtained (particularly for the ABC complex species) were slightly poorer than those obtained with competitive spectroscopic techniques such as mass and infrared spectroscopy. However, they were satisfactory overall. It should be noted that the present study encompassed a high number of *Acinetobacter* species, which indeed have never been considered in the literature for similar studies. Such a large collection certainly embraces high bacterial intra- and inter-species diversity which could have contributed to a decrease in the overall performance of the approach. The greater the number of species involved, the greater the difficulty of discriminating them with a high success rate. This work could have benefited from additional studies in order (i) to better understand the features underlying *Acinetobacter* species discrimination by Raman spectroscopy and (ii) to clarify the clustering dissimilarity found between Raman data and the results obtained from the core genome-based tree of the *Acinetobacter* genus presented by Nemec et al. The standardization of techniques regarding culture media, time of growth and/or equipment setting related parameters such as excitation wavelength, laser power and time of exposure, as well as increasing the number of isolates of each *Acinetobacter* species, could contribute to such a purpose.

## Figures and Tables

**Table 1:** Details of the first PLSDA model developed for *Acinetobacter* species discrimination.

PLSDA Model	Spectra Pre-Processing *	Number of Latent Variables (LVs)	Clusters	Species	% of Correct Assignments
			A	*A. baumannii*	
				*A. calcoaceticus*	
				*A. pittii*	
				*A. nosocomialis*	
				*A. seifertii*	
I		5		*A. dijkshoorniae*	94.3%
			B	*A. haemolyticus*	
				*A. courvalinii*	
				*A. beijerinckii*	
				*A. gyllenbergii*	
				*A. modestus*	
				*A. proteolyticus*	
				*A. venetianus*	
				*A. vivianii*	
				*A. dispersus*	
				*A. colistiniresistens*	

* After pre-processing spectra were always mean-centered.

**Table 2:** Details of PLSDA models developed for Cluster A *Acinetobacter* species discrimination.

PLSDA Model	Spectra Pre-Processing *	Number of LVs	Clusters **	Species	% of Correct Assignments
II-cluster A isolates	---	6	1	*A. dijkshoorniae*	94.4%
			AI	*A. baumannii*	
				*A. calcoaceticus*	
				*A. pittii*	
			AII	*A. nosocomialis*	
				*A. seifertii*	
III-cluster AI isolates	---	5	1	*A. baumannii*	72.1%
			2	*A. calcoaceticus*	
			3	*A. pittii*	
IV-cluster AII isolates	---	5	1	*A. nosocomialis*	87.4%
			2	*A. seifertii*	

* After pre-processing spectra were always mean-centered. ** Clusters identified with letters plus roman numerals always contain two or more species and were subsequently modelled to full species discrimination.

**Table 3:** Details of the PLSDA models developed for Cluster B *Acinetobacter* species discrimination.

PLSDA Model	Spectra Pre-Processing *	Number of LVs	Clusters **	Species	% of Correct Assignments
V-cluster B isolates	Standard normal variate (SNV) + Savitzky-Golay (SavGol) (7,2,1)	6	1	*A. courvalinii*	95.5%
			2	*A. haemolyticus*	
			3	*A. colistiniresistens*	
			BI	*A. dispersus*	
				*A. modestus*	
				*A. venetianus*	
				*A. vivianii*	
				*A. gyllenbergii*	
				*A. proteolyticus*	
				*A. beijerinckii*	
VI-cluster BI isolates	---	4	BI-A	*A. dispersus*	94.2%
				*A. modestus*	
				*A. beijerinckii*	
				*A. gyllenbergii*	
			BI-B	*A. proteolyticus*	
				*A. venetianus*	
				*A. vivianii*	
VII-cluster BI-A isolates	SNV + SavGol (7,2,1)	5	1	*A. dispersus*	98.7%
			BI-AII	*A. modestus*	
				*A. beijerinckii*	
				*A. gyllenbergii*	
VIII-cluster BI-AII isolates	---	4	1	*A. modestus*	81.8%
			2	*A. beijerinckii*	
			3	*A. gyllenbergii*	
VIX-cluster BI-B isolates	SNV + SavGol (7,2,1)	4	1	*A. proteolyticus*	88.1%
			2	*A. venetianus*	
			3	*A. vivianii*	

* After pre-processing spectra were always mean-centered. ** Clusters identified with letters plus roman numerals always contain two or more species and were subsequently modelled to full species discrimination.

## Supplementary Materials

The following are available online, Table S1: *Acinetobacter* strains used in this work [28,29].
