# Supplementary material for: Differentiation of Taxonomically Closely Related Species of the Genus Acinetobacter Using Raman Spectroscopy and Chemometrics
Source: Molecules. 2019 Jan 4;24(1):168. doi: 10.3390/molecules24010168 (PMC6337300; doi:10.3390/molecules24010168)
Supplement: Supplementary file 1 [file molecules-24-00168-s001.pdf]

Table S1. *Acinetobacter* strains used in this work.

Species	Strain no.	Specimen	Locality and date of isolation	Reference
A. baumannii	NIPH 80	I. V. cannula	Praha, CZ, 1993	[12]
	NIPH 146	Wound	Praha, CZ, 1993	
	NIPH 201	Nasal swab	Liberec, CZ, 1992	
	NIPH 329	Tracheal secretion	Tábor, CZ, 1994	
	NIPH 335	Sputum	Tábor, CZ, 1994	
	NIPH 410	Cannula	Brno, CZ, 1996	
	NIPH 527 (= RUH 875)	Urine	Dordrecht, NL, 1984	
A. beijerinckii	NIPH 770	Soil (footpath) Pilion	Peninsula, GR, 1993-1994	[28]
	NIPH 838^{T}	Wound (human)	Malmö, SE, 1980s	
	NIPH 1065	Toe-web (human)	Cologne, DE, 1994	
	NIPH 1453	Airsacculitis (horse)	Gent, BE, 2000	
	NIPH 2014	Throat (human)	Rotterdam, NL, 1988	
	NIPH 2015	Gall (human)	Rotterdam, NL, 1988	
A. calcoaceticus	NIPH 13	Burn	Praha, CZ, 1991	[12]
	NIPH 2155 (= LMG 10516)	Soil	Rotterdam, NL, 1984	
	NIPH 2253 (= LMG 10515)	I. V. cannula	Nijmegen, NL, 1984	
	NIPH 2254 (= LMG 10517)	Wound	Malmö, SE, 1980s	
	NIPH 2262 (= LMG 10518)	Wound	Malmö, SE, 1980s	
	NIPH 2706	Sputum	Pardubice, CZ, 2006	
	NIPH 2814 (= LUH 9144)	Urinary catheter	Leiden, NL, 2004	
	ANC 3801 (= LUH 11899)	Eye	Leiden, NL, 2006	
A. colistiniresistens	NIPH 239	Sputum (human)	Příbram, CZ, 1994	[15]
	NIPH 287	Blood (human)	Příbram, CZ, 1994	
	NIPH 378	Eye (human)	Sedlčany, CZ, 1995	
	NIPH 637	Blood (human)	Praha, CZ, 1995	
	NIPH 669	Blood (human)	Český Krumlov, CZ, 1997	
	NIPH 924	Blood (human)	České Budějovice, CZ, 1998	
	NIPH1035	Blood (human)	Příbram, CZ, 1998	
	NIPH 2036^{T}	Catheter	BE, before 1990	
A. courvalinii	NIPH 1481	Tracheal aspirate (human)	2000	[14]
	NIPH 1847	Conjunctiva (human)	BR, before 1990	
	NIPH 1850	Unknown	Unknown	
	ANC 3930	Wound (human)	Ostrava, CZ, 2011	
	ANC 4230	Blood (human)	Tønsberg, NO, 2006	
	ANC 4930	Urine (human)	Petrovice, CZ, 2014	
	ANC 5178	Shank (human)	Příbram, CZ, 2015	
A. dijkshoorniae	NIPH 814 (= RUH 53)	Flower bed soil	Before 1961	[11]

Species	Strain no.	Specimen	Locality and date of isolation	Reference
	NIPH 2230 (= LUH 7351)	Nephrology drain (human)	Leiden, NL, 2001	
	NIPH 2240 (= ACI 749)	Tracheal aspirate (human)	IT	
	ANC 4052	Blood (human)	USA, 1995-2002	
	ANC 5637^{T} (= JVAP01^{T})	Urine (human)	TR, 2009	
A. dispersus	NIPH 1867	Ulcer (human)	FR, before 1990	[14]
	NIPH 1868	Wound (human)	FR, before 1990	
	NIPH 2020	Sewage water	Taastrup, DK, 1997	
	ANC 3884	Swab	Ostrava, CZ, 2010	
	ANC 4547	Well water (garden)	Lišov, CZ, 2013	
	ANC 4651	Soil (forest creek bank)	Křivoklátsko forestland, CZ, 2013	
A. gyllenbergii	NIPH 230	Vagina (human)	Praha, CZ, 1994	[28]
	NIPH 822	Wound (human)	FR	
	NIPH 1773	Tracheal aspirate (human)	Hong Kong, CN, 1998	
	NIPH 2021	Sinus (human)	Leiden, NL, 2000	
	NIPH 2022	Sputum (human)	Rotterdam, NL, 1989	
	NIPH 2150^{T}	(human)	Leiden, NL, 1978	
	NIPH 2353	Throat (human)	CZ	
A. haemolyticus	NIPH 58	Blood (human)	Praha, CZ, 1992	[14]
	NIPH 261	Tissue (human)	České Budějovice, CZ, 1993	
	NIPH 326	Vaginal swab (human)	Tábor, CZ, 1994	
	NIPH 510^{T}	Sputum (human)	DE, before 1962	
	NIPH 1873			
	NIPH 1874		Malmö, SE, 1980s	
	NIPH 1877			
	NIPH 1878	Wound (human)	Malmö, SE, 1980s	
A. modestus	NIPH 236^{T}	Urine (human)	Příbram, CZ, 1993	[14]
	NIPH 972	Blood (human)	Rotterdam, NL, 1981	
	NIPH 2375	Sewage water	Glostrup, DK, 1997	
	ANC 3862	Wound (human)	Ostrava, CZ, 2010	
	ANC 4109	Sludge (pond dam)	Ortvínovice, CZ, 2011	
	ANC 4229	Blood (human)	Drammen, NO, 2007	
A. nosocomialis	NIPH 386	Sputum	Příbram, CZ, 1996	[12]
	NIPH 523 (= ATCC 17903)	Not known	Before 1950	
	NIPH 2117 (= LMG 10622)	Bronchus	Rotterdam, NL, 1987	
	NIPH 2120 (= LMG 10617)	Skin	Rotterdam, NL, 1987	
	NIPH 2121 (= LMG 10623)	Rectum	Rotterdam, NL, 1987	
	NIPH 2134 (= LMG 10620)	Urine	Nijmegen, NL, 1984	
	NIPH 2265 (= LMG 10626)	Urine	Malmö, SE, 1980s	
A. pittii	NIPH 76	Urine	Praha, CZ, 1992	[12]

Species	Strain no.	Specimen	Locality and date of isolation	Reference
	NIPH 95	Urine	Praha, CZ, 1993	
	NIPH 336	Urine	Tábor, CZ, 1993	
	NIPH 789 (= LUH 3538)	Trachea	Debrecen, HU, 1994	
	NIPH 2133	Bronchus	Nijmegen, NL, 1984	
	NIPH 2135 (= LMG 10554)	Drain	Nijmegen, NL, 1984	
	NIPH 2136 (= LMG 10562)	Urine	Rotterdam, NL, 1984	
	NIPH 2141 (= LMG 10561)	Not known	Basel, CH, 1984	
	NIPH 809^{T}	Ear (human)	USA, before 1984	
	NIPH 1959	Blood (human)	Nottingham, UK, 2000	
A. proteolyticus	ANC 3839	Wound (human)	Příbram, CZ, 2009	[14]
	ANC 3849	Wound (human)	Ostrava, CZ, 2010	
	ANC 3924	Wound (human)	Ostrava, CZ, 2010	
	ANC 3928	Wound (human)	Ostrava, CZ, 2010	
	NIPH 806	Throat swab (human)	Rotterdam, NL	
	NIPH 826	Blood (human)	DK, 1990-1991	
	NIPH 1777	Tracheal aspirate (human)	Hong Kong, CN, 1997	
	NIPH 1779	Blood (human)	Hong Kong, CN, 1997	
A. seifertii	NIPH 1780	Hospital environment	Hong Kong, CN, 1997	[13]
	NIPH 1781	Soil	Hong Kong, CN, 1998	
	NIPH 1782	Blood (human)	Leiden, NL, 1993	
	ANC 4045	Blood (human)	USA, 1995-2002	
A. venetianus	NIPH 1924	Oil in a lagoon	Adriatic Sea, Venice, IT, before 1998	
	NIPH 1925^{T}	Seawater	Tel Baruch, IL, before 1972	
	NIPH 1926	Seawater	Japanese Sea, JP, before 1997	[29]
	NIPH 1933	Vegetable market	Hong Kong, CN	
	NIPH 2310	Aquaculture pond	DK	
A. vivianii	NIPH 758	Soil (beetroot field)	Stommeln, DE, 1993-1994	
	NIPH 761	Soil (rye field)	Stommeln, DE, 1993-1994	[14]
	NIPH 776	Soil (vineyard)	Tosi, IT, 1993-1994	
	NIPH 2168^{T}	Clinical specimen	Leiden, NL, before 1990	

NIPH and ANC, strain designation used by the Laboratory of Bacterial Genetics; ^{T}, type strain.
Country abbreviations: BE, Belgium; CH, Switzerland; CN, China; CZ, Czech Republic; DE, Germany; DK, Denmark; FR, France; GR, Greece; HU, Hungary; IL, Israel; IT, Italy; JP, Japan; NL, the Netherlands; NO, Norway; SE, Sweden; TR, Turkey; UK, United Kingdom; USA, United States of America; Blank field, unknown.
